# Effect of particle volume fraction on the settling velocity of volcanic ash particles: insights from joint experimental and numerical simulations

**DOI:** 10.1038/srep39620

**Published:** 2017-01-03

**Authors:** Elisabetta Del Bello, Jacopo Taddeucci, Mattia de’ Michieli Vitturi, Piergiorgio Scarlato, Daniele Andronico, Simona Scollo, Ulrich Kueppers, Tullio Ricci

**Affiliations:** 1 Istituto Nazionale di Geofisica e Vulcanologia, Sezione di Roma 1, Via di Vigna Murata 605, 00143, Roma, Italy; 2 Istituto Nazionale di Geofisica e Vulcanologia, Sezione di Pisa, Via della Faggiola 32, 56126 Pisa, Italy; 3 Istituto Nazionale di Geofisica e Vulcanologia, Osservatorio Etneo, Sezione di Catania, Piazza Roma 2, 95125 Catania, Italy; 4 Ludwig-Maximilians-Universität (LMU), Physical and Experimental Volcanology, Theresienstr. 41/III, 80333 Munich, Germany

## Abstract

Most of the current ash transport and dispersion models neglect particle-fluid (two-way) and particle-fluid plus particle-particle (four-way) reciprocal interactions during particle fallout from volcanic plumes. These interactions, a function of particle concentration in the plume, could play an important role, explaining, for example, discrepancies between observed and modelled ash deposits. Aiming at a more accurate prediction of volcanic ash dispersal and sedimentation, the settling of ash particles at particle volume fractions (*ϕ*_*p*_) ranging 10^−7^-10^−3^ was performed in laboratory experiments and reproduced by numerical simulations that take into account first the two-way and then the four-way coupling. Results show that the velocity of particles settling together can exceed the velocity of particles settling individually by up to 4 times for *ϕ*_*p*_ ~ 10^−3^. Comparisons between experimental and simulation results reveal that, during the sedimentation process, the settling velocity is largely enhanced by particle-fluid interactions but partly hindered by particle-particle interactions with increasing *ϕ*_*p*_. Combining the experimental and numerical results, we provide an empirical model allowing correction of the settling velocity of particles of any size, density, and shape, as a function of *ϕ*_*p*_. These corrections will impact volcanic plume modelling results as well as remote sensing retrieval techniques for plume parameters.

Volcanic ash is injected in the atmosphere during explosive eruptions and, dispersed by wind and eventually deposited on the ground, may cause health, social, and economic disruption during and after an eruption^1^. Where, when, and how ash from an eruption may impact human activities is currently forecast by using numerical simulations of ash dispersal. Current simulations incorporate wind advection, atmospheric diffusion, particle aggregation, and simplified sedimentation models that consider the terminal velocity of particles as if settling individually^2^,^3^. To overcome this last simplification, here we describe joint experimental and numerical simulations investigating the effect of particle volume fraction on the settling velocity of volcanic ash particles in suspensions.

Complexities of the volcanic cloud-atmosphere system and large variations in the size, density, shape and concentration of erupted particles result in large variations in ash settling dynamics and corresponding settling velocity. The terminal settling velocity of a single volcanic ash particle is usually derived analytically and/or experimentally, and previous studies focussed primarily on the effect of particle size, shape, and density, and of atmospheric properties on this parameter^4^,^5^,^6^,^7^,^8^,^9^,^10^,^11^,^12^. This approach considers the settling of an individual particle in a still fluid, ignoring the presence of neighbouring particles. Similarly, the effect of particle volume fraction (*ϕ*_*p*_) on the settling velocity of individual (i.e., not aggregated) particles is not included in most numerical models of ash dispersal^13^,^14^,^15^,^16^,^17^,^18^,^19^,^20^ which are mostly concerned with the fate of volcanic ash in the dilute, medium-distal regions of the plume. These models assume particles are only affected by the drag due to the local velocity of the carrier flow (one-way coupling), ignoring the effect of particle motion on the flow itself (two-way coupling) and inter-particle collisions (four-way coupling). This approach is justified as long as *ϕ*_*p*_ is less than 10^–6^, below which negligible alteration to the structure of turbulence by particles occurs^21^,^22^. With increasing *ϕ*_*p*_ in the fluid, however, two-way and four-way coupling effects are expected to play an increasingly important role on the settling velocity of particles^23^,^24^,^25^,^26^,^27^,^28^. Within a few hundreds of kilometres from the vent, *ϕ*_*p*_ in the plume can be as high as 10^−5^, and up to 10^−3^ in more proximal regions^29^,^30^,^31^. The observations and modelling of localized regions of instability, including particle-rich ‘fingers’ or ‘scalloped umbrella’^32^,^33^, suggest that the dynamics of particle sedimentation is strongly governed by several factors mostly related to particle concentration^34^.

Only very recently has the effect of other volcanic particles perturbing the surrounding fluid-particle system been addressed by numerical simulations of the eruption plume^35^,^36^, but has never been corroborated experimentally. If not calibrated against experimental data on volcanic particles, numerical models may not capture this effect properly, resulting in incorrect estimates of ash depletion rate (or residence time) from the plume. The only experimental studies of gas-particle mixtures linking settling velocity to *ϕ*_*p*_ are found within the engineering literature and involve industrial droplets and particles at the ten micron scale^26^,^28^. These studies reported an increase in the mean particle settling velocity in the mixture compared to the terminal velocity of individual particles in still fluid, explaining this outcome in terms of the preferential sweeping of the particles downstream within local vortical structures^26^,^28^.

With the objective of filling the lack of experimental studies on natural volcanic particles, we have performed a systematic experimental study on the effect of *ϕ*_*p*_ on the settling velocity of volcanic ash particles in suspensions 10^−7^ < *ϕ*_*p*_ < 10^−3^. We also performed numerical simulations that reproduced the experiments using both a two-way and a four-way coupling approach, pointing to: i) identify the key physical processes in place during the experiment, and ii) test the ability of the code to capture the coupling dynamics at the laboratory scale. Finally, we derived an empirical relation that links the main particle characteristics (particle size, density and partly shape) and settling velocity to *ϕ*_*p*_.

## Methods

### Experiments

#### Starting material

**Figure 1:**
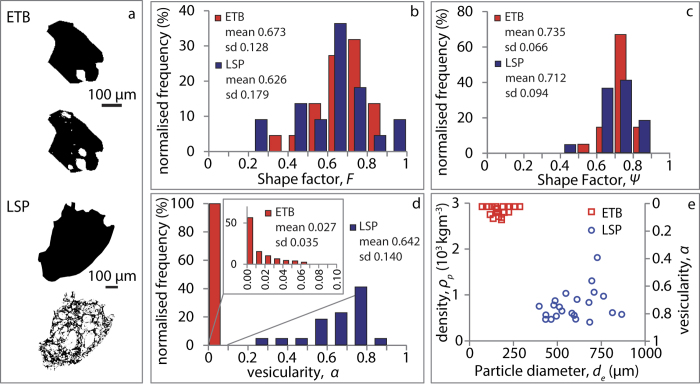
Size, shape, vesicularity and density distribution of the Etna Basalt (ETB), and Laacher See phonolite (LSP) ash samples obtained from SEM analysis. (**a**) Examples of SEM images of ETB and LSP ash particles. The top images represent the particle outline; the bottom images show the area occupied by vesicles (in white). (**b,c)** Normalised frequency distribution of the shape factors *F*^4^ and *Ψ*^10^, used to compute the individual settling velocity of the particles using literature models. (**d**) Frequency distribution of 2-D vesicularity α, calculated as the fraction of the total projected area occupied by voids. Inset shows expanded vesicularity plot for ETB. (**e**) Diameter *de* vs. density *ρ*_*p*_ (and vesicularity *α*) diagram. The density of the analysed particle population is calculated from bulk density according to the vesicularity of each particle.

Two volcanic ash samples differing in composition, density, shape and size distributions were used in our experiments: i) dense basaltic ash from Etna (ETB), sieved in the diameter classes 125 μm < *d* < 500 μm; and ii) pumiceous phonolitic ash from Laacher See (LSP) sieved in the class 500 μm <*d* <1000 μm. Shape and vesicularity of the starting material were characterized on a population of approximately 50 clasts by Scanning Electron Microscope (JEOL JSM-6500F) images analysis (Fig. 1). For each particle, binary images generated by thresholding the total projected area and the area fraction occupied by voids, were analysed using the *ImageJ* analysis toolbox, obtaining the following parameters: i) projected area, ii) perimeter, iii) major and iv) minor axis, v) void fraction. The shape parameters *F*^4^ and *Ψ*^10^ (definition in Supplementary Methods S1), calculated assuming the particles as prolate spheroids, show mean values of 0.67 and 0.74 and 0.63 and 0.71 for ETB and LSP, respectively (Table 1). Vesicularity *α* (i.e. the area fraction occupied by voids) is always negligible (*α* < 0.1) for ETB samples, with 56% of the analysed population showing *α* < 0.01. Vesicularity varies significantly for LSP, spanning from 0.3 to 0.9 with ca. 60% of the analysed samples showing *α* > 0.7 (Fig. 1).

Density of the solid fraction (*ρ*_*s*_, including closed pores) was measured using a helium pycnometer (Micrometrics AccuPyc II 1340), obtaining 2925 ± 32 kg/m^3^ and 2136 ± 41 kg/m^3^ for ETB and LSP, respectively. The density of the analysed particles was computed as *ρ*_*p*_ = *ρ*_*S*_(1−*α*), assuming that measured 2-D vesicularity is representative of the actual 3-D vesicularity of the bulk particle. Mean density values of the analysed ETB and LSP samples are 2846 ± 101 kg/m^3^ and 764 ± 298 kg/m^3^, respectively, equal to vesicularity of approximately 3 and 65%. The average density of about 3 × 10^4^ LSP particles was also measured by an independent method^37^, obtaining a value of 831 ± 60 kg/m^3^.

#### Experimental setup

**Figure 2:**
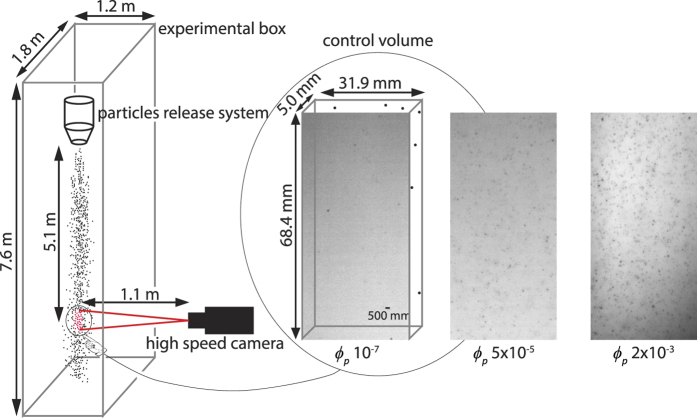
Schematic diagram of the experimental setup. Particles are released at variable discharge rates within an experimental box open at the top and bottom, and filmed at 2000 frames per second while settling. The camera focuses on a small control volume at the center of the stream of settling particles. Small flow width (~0.1 m) with respect to box size minimizes wall effects. Still frames on the right show examples of different particle volume fraction (*ϕ*_*p*_) within the control volume (see Supplementary Video S2).

The experiments consisted of the release of a vertical flow of particles in a box, open at the top and bottom, with sizes 7.6 m (height) × 1.8 m (width) × 1.2 m (depth), at ambient pressure (1016 ± 2 hPa), temperature (27 ± 1 °C) and relative humidity (54 ± 3%) conditions (Fig. 2). For both ETB and LSP, different experimental runs were conducted by varying the mass discharge rate at the flow source from a maximum of 1.14 × 10^−1^ kg/s (run f1) to a minimum of 2 × 10^−4^ kg/s (run f5) using parcels of the same starting material (i.e., same grain size, shape, and density distributions). The resulting experimental durations ranged from a maximum of 176 s to a minimum of 10 s. A high-speed camera (Optronis CR600 × 2) equipped with a 135 mm lens was used to acquire images of the particles (at 2000 Hz and 114 μm pixel resolution) in a small region, or control volume, at the centre of the stream of settling particles. This control volume of size 68.4 mm × 31.9 mm × 5 mm was defined by the camera field of view multiplied by the depth of field and was located 5.1 m below the release system.

#### Image processing & data analysis

A custom designed routine (PyTV^38^) was used to track frame-by-frame each particle in the imaged area of the flow. For each point of the track, the routine provided the x-y position and the projected area of the tracked particle (see Supplementary Video S3). Due to particle interaction during the settling (see below), particle volume fraction varied during each experimental run, as observed in similar literature experiments^26^. Thus, for each experimental run, a 50 frames-long (i.e. 0.025s) bin was sampled every 5s starting from the appearance of the first particles to the end of the experiment. Each bin contained a variable number of tracks ranging ca. 2 to 9 × 10^2^. The routine tracked ca. 1.1 × 10^4^ particles (sum of all runs), from which the following, track-averaged parameters were obtained: i) equivalent particle diameter *d*_*e*_, taken as the diameter of an equivalent sphere with the same projected area; ii) settling (vertical) velocity *v*_*p*_. For each bin, we also calculated the particle volume fraction *ϕ*_*p*_ as the sum of the volumes of all particles in one frame (calculated as *V* = 4/3*π*(*d*_*e*_/2)^3^) divided by the control volume. The obtained *ϕ*_*p*_ is a lower bound, considering that we confidently tracked about 90% of the particles within the control volume. In addition, the frame by frame position and area of 20–50 particles per run (using ImageJ Software, with *MTrakJ* plug-in extension) was obtained manually to check consistency of the parameters obtained with the tracking routine.

Results are compared with the models of Kunii and Levenspiel^5^, Wilson and Huang^4^, and Dellino *et al*.^10^ for the settling velocity of (both volcanic and non-volcanic) individual particles in still fluids (described in Supplementary Methods S1), computed using *d*_*e*_, mean density, and shape factor values of starting material particles as described above.

### Numerical Simulations

An Eulerian-Lagrangian approach was adopted to numerically simulate the settling of particles in our ETB experiments, by using the MPPICFoam OpenFoam solver (based on the MultiPhase Particle-in-Cell method), which includes the effect of the particle volume fraction on the continuous phase. A detailed description of the modelling approach used and the set of underlying equations can be found in the Supplementary Methods S1. The solver has been used to simulate the release and the fall of particles in a domain closed on the sides and open at the bottom with the same size of the experimental box. For each simulation, about 10^6^ to 10^7^ solid spherical particles are released from the top of the domain from a circular vent with a fixed diameter (0.019 m) and initial null velocity. Three different mass discharges have been investigated: i) a total mass of 1.1 kg for 10 s (1.1 × 10^−1^ kg/s), ii) a total mass of 1.24 kg for 40 s (3.1 × 10^−2^ kg/s), iii) a total mass of 0.0352 kg for 176 s (2.0 × 10^−4^ kg/s), reproducing the experimentally generated maximum (f1), intermediate (f3) and minimum (f5) mass discharge rate, respectively. Particles density is assumed constant (2600 kg/m^3^) and the diameter is described by a truncated normal distribution with mean value of 300 μm, variance 100 μm and minimum and maximum diameter fixed at 100 and 400 μm respectively, similar to the ETB case. For each mass discharge rate, two types of simulations were performed: one with a 2-way coupling (S2W), where the reciprocal drag of the interacting air and particles is considered, and one with a four-way coupling (S4W), where also the collisions between particles are accounted for. Similarly to the experiments, the mean particle volume fraction, velocity, and diameter data of the simulated particles were sampled every 0.2 s within a control volume located 5 m below the release source (Table 1).

## Results

**Figure 3:**
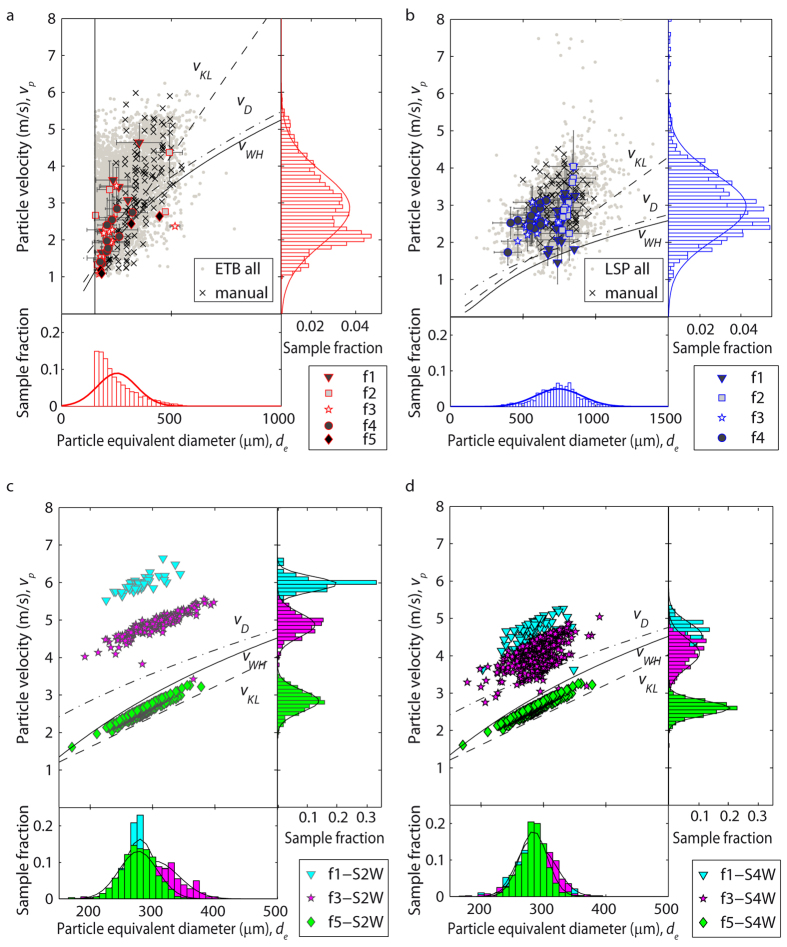
Size (*d*_*e*_) and settling velocity (*v*_*p*_) of (**a**) particles from Etna basalt (ETB) and (**b**) Laacher See phonolite (LSP) experiments, and (**c**) the two-way (S2W) and (**d**) four-way (S4W) numerical simulations. The theoretical variation of settling velocity with size is also plotted using the models of Wilson and Huang (*v*_*WH*_)^4^, Kunii and Levenspiel (*v*_*KL*_)^5^ and Dellino *et al. (v*_*D*_)^10^. The bottom and lateral panels in each sub-figure show the frequency distribution and normal fits of *d*_*e*_ and *vp*, respectively (note different scales). In (**a**) and (**b**), black crosses and grey dots represent manual and automatic tracking data respectively, while filled symbols denote the mean value with standard deviation (error bar) of data binned (every 5 s) for different mass discharge rate runs (f1, maximum, to f5, minimum). The vertical line in (**a**) indicates the image resolution limit. In (**c**) and (**d**), data points represent the mean value sampled every 0.2 s of simulation time for the different mass discharge rate runs (different symbols).

The settling velocity (*v*_*p*_) of particles measured in both the ETB and LSP experiments spans between 1 and 6 m/s, with similar modal values around 2.5–3 m/s, and a rather platykurtic Gaussian distribution for the narrow range of particle sizes used. Automatic and manually tracked particle velocities plot in the same range (Fig. 3, Table 1). As expected, *v*_*p*_ increases with particle size (*d*_*e*_). For any particle size, the vast majority of the measured velocities are higher than those predicted from models in the literature, which only approximate well the lowest measured velocities. For any particle size, runs with higher discharge rate tend to display higher velocities for the ETB sample (see Supplementary Video S2), while this trend is not evident for LSP.

The numerical simulations of ETB (Supplementary Video S4–S5) match well the range of experimentally observed velocities. For the minimum discharge rate (f5), a good correlation with the literature models of Kunii and Levenspiel^5^ and Wilson and Huang^4^ is also observed, while the correlation is worse when using the model of Dellino *et al*.^10^, developed for larger and more vesicular clasts and already known to overestimate the settling velocity of particles smaller than ~500 μm^2^,^6^. For the intermediate (f3) and maximum (f1) discharge rates, velocities from the numerical simulations are always higher than those predicted by previous literature models considering the settling of individual particles. With respect to the two-way simulations, at the intermediate (f3) and maximum discharge rates (f1) the settling velocity distributions for the four-way simulations display lower modal values and partial overlapping, thus more closely resembling the experimental results.

**Figure 4:**
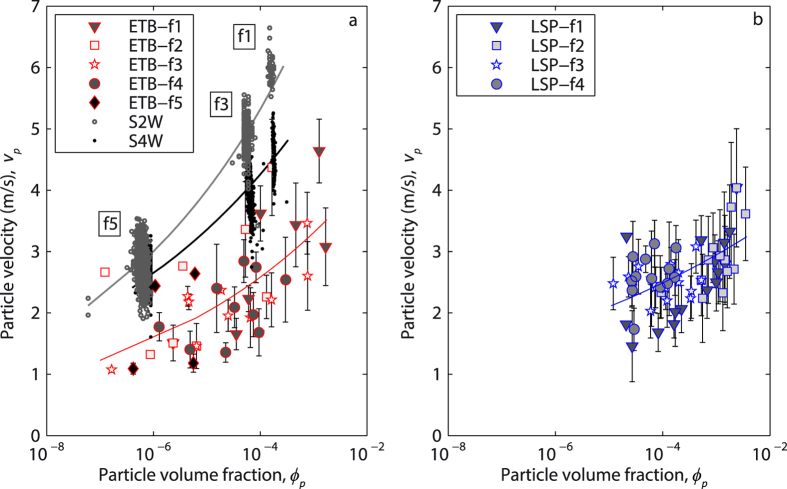
Effect of particle volume fraction (*ϕ*_*p*_) on the settling velocity of particles (*v*_*p*_) for the Etna Basalt (ETB, **a**) and Laacher See phonolite (LSP, **b**) experiments. Filled symbols denote the mean value with standard deviation (error bar) of data binned (every 5 s) for different mass discharge rate runs. The two-way (S2W) and four-way (S4W) numerical simulations are shown for comparison in (**a**), points representing the mean value sampled every 0.2 s of simulation time for the different mass discharge rate runs. Power-law fit curves for the four data sets are also shown (RMSE of 0.66, 0.45, 0.32, and 0.30 for ETB, LSP, S2W, and S4W, respectively).

More than discharge rate, local particle volume fraction *ϕ*_*p*_ is the main factor enhancing particle settling velocity *v*_*p*_ (Fig. 4). A power law can fit the dependence of these two parameters in both experimental and numerical cases, with *vp* increasing by a factor of 3 when *ϕ*_*p*_ increases from 2 × 10^−7^ to 2 × 10^−3^. With respect to the experimental, irregular particles, the numerical, spherical ones display – as expected – higher velocities for any volume fraction (despite their slightly lower density), higher for the two-way case than for the four-way one, which better parallels the experimental data. Moreover, the numerical simulations results display a good match between the discharge rate used for the different runs and the corresponding particle volume fraction, while this match is less evident for the ETB runs and not observed in the LSP ones, outlining the greater variability of the natural starting material with respect to the numerical model. Note that, both in the ETB and LSP cases, the experimentally-derived settling velocities for the lowermost particle volume fractions match well the settling velocity predicted by literature models for individual particles of the same size.

**Figure 5:**
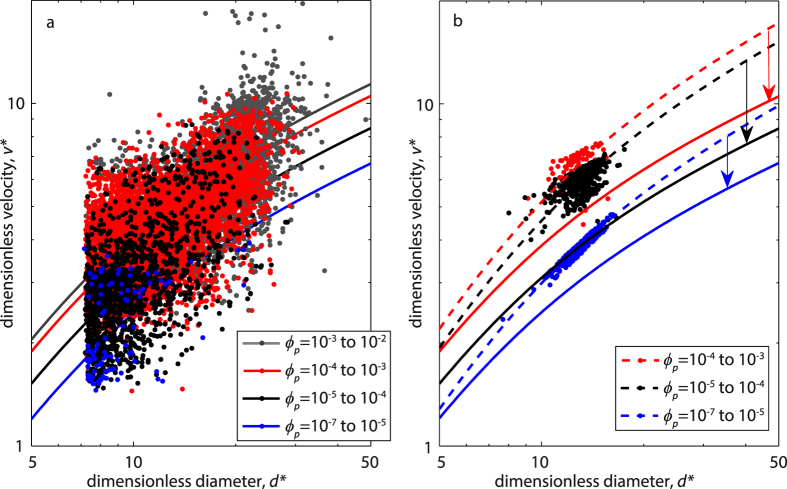
Dimensionless particle size versus dimensionless settling velocity for the experimental (both ETB and LSP, **a**) and numerical (S4W) cases (**b**). Different colors denote particles settling within variable intervals of particle volume fraction (*ϕ*_*p*_, note the larger interval for the lowest *ϕ*_*p*_ values, due to the occurrence of fewer particles in this range). Fitting curves (solid and dashed for the experimental and numerical cases, respectively) for the different *ϕ*_*p*_ intervals follow equation (3). Arrows in (**b**) mark the deviation between spherical (numerical) and irregular (experimental) particles.

Our final goal is to obtain an empirical fit allowing one to calculate the settling velocity of ash particles of known size, density and shape as a function of particle volume fraction. To this goal we followed an approach similar to that of Haider and Levenspiel^12^. First we non-dimensionalized the settling velocity and particle size to compare the experimental (ETB and LSP) and the four-way numerical results (which, compared to the two-way ones, are more realistic and approximate better the experimental results) as:


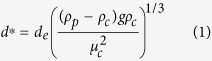



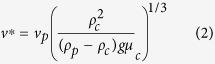


where *μ*_*c*_ and *ρ*_*c*_ are the density and viscosity of the carrier fluid (air) at ambient conditions, respectively, and *g* is gravitational acceleration. All data are then grouped for different *ϕ*_*p*_ intervals and each group is fitted with an empirical expression similar (but not identical) to that used by Haider and Levenspiel^12^ for the settling velocity of spherical and nonspherical particles (Fig. 5)


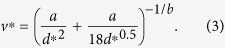


**Figure 6:**
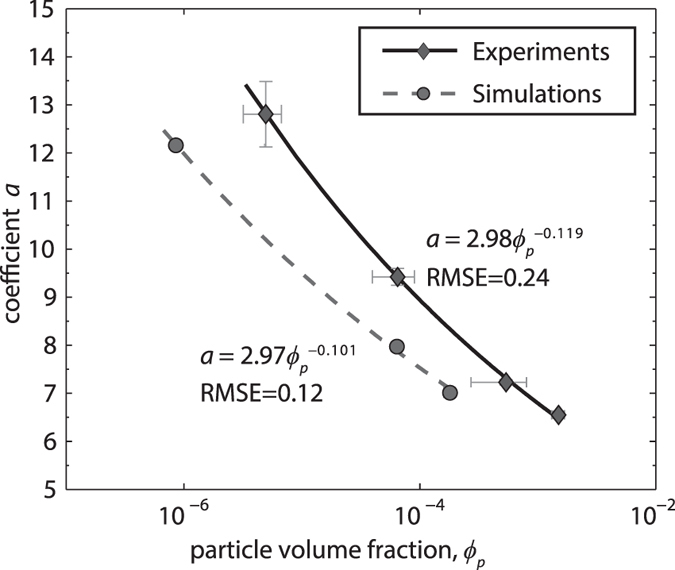
Power law dependence of the fit coefficient *a* to particle volume fraction for the experimental data and numerical simulations. Error bars are the standard deviation of the average volume fraction and the 95% confidence intervals of coefficient *a*.

Given that the use of dimensionless parameters accounts for particle size and density, the coefficients *a* and *b* of the fit curves are, in our case, function of particle shape and volume fraction only. The goodness of fit given by Equation (3), expressed by the Root Mean Square Error (RMSE), is quite high for the numerical case (RMSE of 0.07–0.62) and more variable for the experimental one (RMSE of 0.60–1.85). We found that fit error is minimized for *b* = 1 and *b* = 1.2 for the spherical particles (numerical case) and the irregular ones (experimental case), respectively. These values are also those that fit better the runs with the lowest particle volume fraction, i.e., those more similar to the case of particles settling individually. Holding the above *b* values constant, we found that *a* decreases as a function of volume fraction following a power law (Fig. 6), with a constant coefficient ~2.9 and an exponent almost equal to −0.1*b* so that Equation (3) can be rewritten as


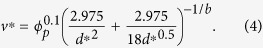


Equation (4) directly links the dimensionless settling velocity and size of particles to the particle volume fraction, with two distinct (but similar) coefficients for spherical and irregular particles, respectively.

## Discussion and Conclusions

Our experiments show that ash-sized natural volcanic particles settle at greater absolute velocity when the particle volume fraction *ϕ*_*p*_ in the surrounding medium increases. The commonly used terminal settling velocity of an individual particle in a still fluid represents the lowest bound for *ϕ*_*p*_ → 0. The rate of settling velocity increase with increasing particle volume fraction is similar for both Etna and Laacher See samples, despite their different particle shape and density.

Particle volume fraction has two impacts on settling velocity. First, the relative motion of an individual particle with respect to the surrounding fluid (two-way coupling) induces turbulence along its trajectory, causing a neighbouring particle to be preferentially swept to the downward side of the eddies, with a resultant net force that accelerates downwards both particles and air^21^. Second, as the particle volume fraction becomes higher, in addition to the two-way coupling between the particles and fluid, particle-particle collisions (four-way coupling) take place increasingly. Such collisions, enhanced by differential settling velocity, may have a horizontal component and do not necessarily result in a downward acceleration. The numerical simulations allow discriminating the effects of two-way and four-way coupling on our experimental results. The S2W case shows the highest settling velocity for any particle volume fraction and is the one that deviates most from the individual particle case. As expected, two-way coupling strongly enhances settling velocity. In the S4W case, this enhancing effect is attenuated by particle collisions, which randomly deviate trajectories and dissipate downward kinetic energy. As a result, at the same particle volume fraction, settling velocity is lower and with a broader distribution with respect to the S2W case. Four-way effects are usually assumed^24^,^25^,^26^ to become important at *ϕ*_*p*_ > 10^−3^, but in our numerical cases, possibly as a result of the relatively broad initial size distribution of particles used, these effects seem to became apparent even at lower values of *ϕ*_*p*_ (Fig. 4). For the lowermost *ϕ*_*p*_ values, the good match between the experimental velocity values and those predicted by literature models^4^,^5^ for particles settling individually suggests that at *ϕ*_*p*_ ~ 10^−7^ two-way effects are still negligible, in agreement with literature observations^24^,^26^. At any *ϕ*_*p*_, the settling velocity distribution of the S4W case replicates the experimental case better than the S2W case (Fig. 3), thus confirming the importance of four-way interactions at the conditions proper for the experimental case. Non-sphericity, surface roughness, and density variations of natural particles account for the larger scatter of the experimental case with respect to the S4W one. The density-induced deviations are accounted for in our dimensionless analysis. Thus, only shape differences cause the deviations (visible in Fig. 5 and quantified in Fig. 6 and through the *b* coefficient) between the ideal, spherical particles and the natural, irregular ones.

For a given particle volume fraction, Equation (4) allows adjusting dimensionless velocity and thus the settling velocity of particles of known size and density. In addition, the two values of the *b* coefficient allow adding, to a limited extent, information on the particle shape. Equation (4) is valid only within our experimental boundaries, i.e., 10^−7^ < *ϕ*_*p*_ < 10^−3^ and 7 < d^*^ < 50, corresponding to 10 < Re < 100, typical for the intermediate conditions (between laminar and turbulent) that characterize the settling of coarse ash from volcanic plumes^11^.

Our results stem from observations at a millimetre-scale, and their applicability on a larger scale could be questionable. However, temporal fluctuations in our observation window, resulting from experimental and intrinsic effects, partly compensate this spatial limitation by exposing different flow conditions to our analysis. The larger scale validity of our experimental results is corroborated by both the numerical results, which are free from undesired experimental artefacts, and by previous experimental results that shows how velocity/volume fraction relationships are consistent in different regions of the flow^26^. Reciprocally, the convergence of results supports the applicability of our numerical methods to capture two- and four-way coupling dynamics. Future research will investigate the role of other factors (e.g., grain size distribution, presence of ash aggregates, ambient temperature, pressure and humidity) on our results.

To our knowledge, our experiments are the first to investigate four-way coupling using natural volcanic particles. Our results show a significant deviation from ideal, spherical particles. The range of particle size and volume fraction used in the models and experiments match well those expected, for instance, inside recent eruption plumes from Mt. Etna (Italy) within 20 km from the eruptive vent^30^,^39^,^40^,^41^. In such a case, an ash accumulation rate on the ground of about one centimeter per hour would translate, assuming particles settling at 1 m/s, to a *ϕ*_*p*_ of about 4 × 10^−6^, with four-way coupling effects starting to be important. Observed accumulation rates of one millimeter per hour^42^ would translate to a *ϕ*_*p*_ of about 10^−7^. However, these *ϕ*_*p*_ values apply in the volume of air immediately above the ground, while at the base or inside the plume *ϕ*_*p*_ is expected to be higher, advection and dispersal forces diluting particles during settling (as seen, on a smaller scale, in our case, see Supplementary Video S5).

Four-way coupling may play an important role in controlling particle sedimentation in different parts of the volcanic plume. Inside the plume itself, in order to sustain particles in suspension, the upward component of turbulence must balance not only the theoretical, quiescent-fluid terminal velocity of individual particles but also the enhanced velocity due to particle interactions. Below the plume, regions of higher particle volume fractions are known to develop, e.g., particle-rich ‘fingers’ or ‘scalloped umbrella’^32^,^33^, and the settling rate from such regions will change according to the local particle volume fraction. In all these cases, our results show the actual velocity of the particles settling at *ϕ*_*p*_ ~ 10^−3^ may reach up to a factor of 3–4 higher than that of individual particles settling in a still fluid. Such an increase in particle settling rate would result in earlier deposition of particles of a given size (and consequent depletion of such particles from the plume), affecting the areal and grain size distributions of eruption deposits. Inside such regions, four-way coupling will also impact particle aggregation and disaggregation processes^43^, and possibly remove finer particles from the plume because of their sweeping by the downdraft of larger ones. Our observations may be also extended to the study of pyroclastic density currents, where the settling velocity of particles is often used to infer the physical properties of the flow^44^. In such currents, *ϕ*_*p*_ is commonly in the range 10^−5^–10^−2^, relevant for four-way coupling. Equation 4 may be a rapid alternative to numerical modelling when accounting for four-way coupling in estimating properties of the currents.

Several plume measurement techniques, including Doppler Radar stations and disdrometers, may use inversion algorithms to determine the grain size of settling particles in atmosphere from the measured velocity^45^,^46^. Such algorithms are based on still-fluid, individual particle theoretical models that neglect the effect of particle volume fraction. In the presence of such effects particle size would be overestimated.

Most numerical models of ash dispersal adopt a one-way coupling approach^13^,^14^,^15^,^16^,^17^,^18^,^19^,^20^, an assumption justified in the dilute regions of the plume where *ϕ*_*p*_ is less than 10^−6 ^31, in agreement with our above statements. However, depending on plume height, *ϕ*_*p*_ may be 10^−5^–10^−6^ in regions within a few hundreds of kilometres and up to 10^−3^ more proximally^29^,^30^,^31^. This range of volume fractions is covered by our experiments, whose results detail for the first time the role of two-way and four-way coupling effects for volcanic particle mixtures. Two-way coupling is being recently incorporated in numerical simulations of volcanic plumes, but it requires new code development and the simultaneous solution of the equations describing the atmospheric fields and those describing the ash particles trajectories, with a big impact on the computational cost^35^,^36^ and the real time applicability during a crisis. Equation (4) could provide an empirical alternative also accounting for four-way effects, with a limited application range as for particle size but easily incorporated into pre-existing codes.

## Additional Information

**How to cite this article**: Del Bello, E. *et al*. Effect of particle volume fraction on the settling velocity of volcanic ash particles: insights from joint experimental and numerical simulations. *Sci. Rep.* **7**, 39620; doi: 10.1038/srep39620 (2017).

**Publisher's note:** Springer Nature remains neutral with regard to jurisdictional claims in published maps and institutional affiliations.

## Supplementary Material

Supplementary Information

Supplementary Video S2

Supplementary Video S3

Supplementary Video S4

Supplementary Video S5

## Figures and Tables

**Table 1:** Statistical parameters of the size, shape, and settling velocity distributions (plotted in Figs 1 and 3) of the natural Etna basalt (ETB) and Laacher See phonolite (LSP) ash particles used in the experiments and of the ideal, spherical particles used in the two-way (S2W) and four-way (S4W) coupling numerical simulations.

Experiments	n° tracked particles	Parameter	Mean	Median	Mode	Percentile (25th)	Percentile (75th)	Skewness	Kurtosis
ETB	8551								
		*d*_*e*_	252.13	223.91	154.36	183.73	297.63	1.26	4.31
		*v*_*p*_	2.86	2.75	1.09	2.10	3.49	0.48	2.68
		*F*	0.67	0.69	0.40	0.61	0.73	−0.55	3.18
		Ψ	0.74	0.75	0.76	0.71	0.76	−0.69	3.95
LSP	2453								
		*d*_*e*_	745.40	745.31	654.90	648.72	833.99	0.43	5.88
		*v*_*p*_	2.97	2.86	2.10	2.36	3.50	1.29	7.52
		*F*	0.63	0.66	0.28	0.51	0.71	−0.34	2.59
		Ψ	0.71	0.72	0.64	0.64	0.78	−0.36	3.78
**Simulations**	**n° analysed samples**								
S2W									
	48	f1 (10s)							
		*d*_*e*_	279.63	277.11	225.80	266.48	290.73	0.54	3.48
		*v*_*p*_	5.96	5.96	5.52	5.85	6.03	0.98	5.35
	182	f3 (40s)							
		*d*_*e*_	303.14	303.13	191.64	274.23	328.96	0.07	2.74
		*v*_*p*_	4.86	4.89	3.42	4.68	5.05	−0.74	5.22
	880	f5 (176s)							
		*d*_*e*_	277.47	277.83	252.15	260.44	295.99	−0.27	3.76
		*v*_*p*_	2.84	2.84	2.75	2.65	3.02	−0.27	3.03
		*F*	1.00	1.00	1.00	1.00	1.00		
		Ψ	1.00	1.00	1.00	1.00	1.00		
S4W									
	95	f1 (10s)							
		*d*_*e*_	282.63	282.86	202.54	267.33	296.64	−0.01	3.66
		*v*_*p*_	4.53	4.54	3.62	4.28	4.81	−0.28	2.71
	397	f3 (40s)							
		*d*_*e*_	291.37	292.51	178.01	273.42	310.76	−0.36	4.03
		*v*_*p*_	4.07	4.12	3.69	3.80	4.38	−0.48	2.91
	705	f5 (176s)							
		*d*_*e*_	284.01	284.62	260.63	270.81	297.81	−0.06	4.42
		*v*_*p*_	2.60	2.61	2.55	2.48	2.72	−0.22	4.18
		*F*	1.00	1.00	1.00	1.00	1.00		
		Ψ	1.00	1.00	1.00	1.00	1.00		
