# Supplementary material for: Effect of particle volume fraction on the settling velocity of volcanic ash particles: insights from joint experimental and numerical simulations
Source: Sci Rep. 2017 Jan 3;7:39620. doi: 10.1038/srep39620 (PMC5206740; doi:10.1038/srep39620)
Supplement: Supplementary Information [file srep39620-s1.pdf]

EFFECT OF PARTICLE VOLUME FRACTION ON THE SETTLING VELOCITY OF VOLCANIC ASH PARTICLES: INSIGHTS FROM JOINT EXPERIMENTAL AND NUMERICAL SIMULATIONS

Elisabetta Del Bello*[1], Jacopo Taddeucci[1], Mattia de' Michieli Vitturi[2], Piergiorgio Scarlato[1], Daniele Andronico[3], Simona Scollo[3], Ulrich Kueppers[4], Tullio Ricci[1]

*Corresponding author. Contacts: elisabetta.delbello@ingv.it, phone no. +39 328.1341102

SUPPLEMENTARY INFORMATION GUIDE

The list of supplementary information files is provided below with a description of their content:

- *Supplementary Methods S1* (included in this pdf file). Contains two sections describing i) previous equations for the settling velocity of individual volcanic particles, and ii) the modelling approach and the set of underlying equations adopted for the numerical simulations.

- *Supplementary Video S2, mov*. Examples of videos recorded at 2000 frames per second during experimental runs with Etna basaltic ash particles (ETB). The three panels show examples of experimental runs conducted at mass discharge rates of 2.0 x 10^{-4} kg/s (f5, left panel), 3.1 x 10^{-2} kg/s (f3, centre panel), 1.1 x 10^{-1} kg/s (f1, right panel). The natural duration of the video is 0.154 s. The average particle volume fraction (ϕ_p) retrieved in these extracts is~10^{-7}, ~5*10^{-5}, ~2*10^{-3}.

- *Supplementary Video S3, mov*. Illustration of the video processing steps. The left panel shows an extract (0.08 sec) of the original video of Etna basaltic ash particles (ETB). The center panel show the resulting pre-processed output of the video obtained by subtracting the dynamic background from the original images. The right panel shows the tracked particles retrieved using the automatic tracking routine (PyTV). Only in-focus particles within the control volume are tracked by the routine.

- *Supplementary Video S4, mov*. This video contains the result of the two-way coupling (particle-fluid interactions) Eulerian-Lagrangian simulations of ETB particles settling. The simulation reproduces the experimental run with discharge rate 1.1 x 10^{-1} kg/s and duration 10s. The left panel illustrate a 3D view of the particles (scale representing particle diameter *d*, in meters). The central and right panels illustrate the particle volume fraction (ϕ_p, dimensionless), and the gas velocity (in m/s) along an axial 2D vertical slice.

- *Supplementary Video S5, mov*. This video contains the result of the four-way coupling (particle-fluid and particle-particle interactions) Eulerian-Lagrangian simulations of ETB particles settling. The simulation reproduces the experimental run with discharge rate 1.1 x 10^{-1} kg/s and duration 10s. The left panel illustrate a 3D view of the particles (scale representing particle diameter *d*, in meters). The central and right panels illustrate the particle volume fraction (ϕ_p, dimensionless), and the gas velocity (in m/s) along an axial 2D vertical slice.

SUPPLEMENTARY METHODS S1

I. PREVIOUS EQUATIONS FOR THE SETTLING VELOCITY OF INDIVIDUAL VOLCANIC PARTICLES

The terminal settling velocity (v_t) of a particle is defined theoretically as the velocity attained when aerodynamic drag balances the accelerating force (gravity). Its study is relevant for a variety of natural particle-laden flow systems in which the discrete phase have a density larger than that of the carrier fluid, like, e.g., ash/rain/hail settling in the atmosphere[1–3], or sediment transport in rivers[4]. Its simplified expression derives from the Navier-Stokes equation of motion in which the inertial force term is neglected, and is widely used in numerical models of ash transport and sedimentation[5]. For an individual particle settling in a still medium, v_t can be expressed as

$$v_t = \sqrt{\frac{4}{3}\frac{(\rho_p - \rho_c)d_p g}{C_D \rho_c}}\,, \tag{1}$$

where $\boldsymbol{g}$ is gravitational acceleration, ρ_p is the particle density, d_p the particle diameter, ρ_c is the air density, and C_D is the drag coefficient. This latter parameter is a complex function of particle properties (including, e.g., size, shape), and of the properties and structure of the carrier fluid and is usually derived empirically.

Regarding volcanic ash, the treatment of Kunii and Levenspiel[6] is widely used to derive a set of different empirical correlations of v_t according to the flow regimes (expressed through the Reynolds number $\mathrm{Re} = \rho_c v_t d_p / \mu_c$, where μ_c is the dynamic viscosity of air) pertinent to the gravitational settling of volcanic ash particles [7,8]. In this treatment, the effect of shape is discarded and the particles are assumed spherical. In the transitional regime (0.4<Re<500) it is expressed as:

$$v_{KL} = d\left(\frac{4\rho^2 g^2}{255\,\mu_c \rho_c}\right)^{\frac{1}{3}} \tag{2}$$

Another widely used model derives v_t from Wilson and Huang[9], where the drag coefficient of differently shaped particles is experimentally defined as a function of Re and the shape parameter $F = (b + c)/2a$ calculated using the particle axial lengths a > b > c:

$$C_D = \frac{24}{\mathrm{Re}} F^{-0.828} + 2\sqrt{1.07 - F}\ , \qquad (3)$$

obtaining v_t from the combination of (1) and (3).

Another experimentally defined expression is based on a more sophisticated particle shape parameter Ψ [10]

$$v_D = \frac{1.2065\,\mu_c (d^3 g(\rho - \rho_c)\rho_c \Psi^{1.6} / \mu_c^2)^{0.5026}}{d\rho_c}\,. \qquad (4)$$

The quantity Ψ represents the ratio of sphericity Φ to circularity χ, either used individually, or combined as shape descriptors[11–14]. Sphericity Φ is defined by the ratio of the surface area of the equivalent sphere (calculated from the equivalent diameter d_e) and the surface area of the actual particle. Circularity χ is the ratio of the particle perimeter and the equivalent circumference of a prolate ellipsoid. Here, equations 1-4 were used to calculate values of v_{WH}, v_{KL}, and v_D respectively, using the measured shape and density data (see section 2) of the particles used in our experiments.

II. EULERIAN-LAGRANGIAN SIMULATIONS OF PARTICLES SETTLING IN A TWO-PHASE FLOW. MODEL DESCRIPTION

When dealing with the movement of a group of particles inside a fluid, there are basically two different ways to approach the problem.

i. In the Eulerian-Eulerian approach, the particles are treated as a continuous phase and conservation equations are solved for the particulate phase. This method is suitable for large particle concentrations, where two-way coupling between the fluid and the particulate phases as well as particle-particle collisions are important.

ii. In the Eulerian-Lagrangian approach, the Eulerian continuum equations are solved for the fluid phase, while Newton's equations for motion are solved for the particulate phase in order to determine the trajectories of the particles (or group of particles).

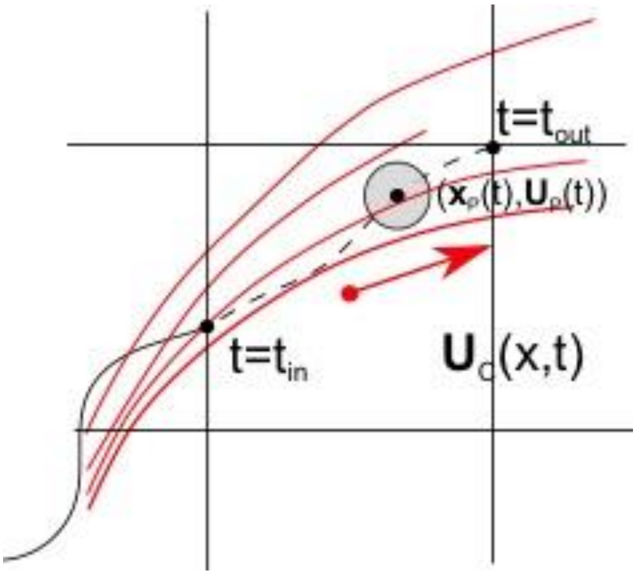

Figure 1. Eulerian-Lagrangian approach adopted for the numerical simulations. The particle *P* is represented by its position $\boldsymbol{x}_p$ and velocity $\boldsymbol{U}_p$, updated solving the Newton's equations for motion. The velocity of the fluid phase is described by the field $\boldsymbol{U}_c$, obtained solving the phase momentum equation on an Eulerian mesh.

The OpenFoam solver adopted, namely MPPICFoam, is based on an Eulerian-Lagrangian approach, and allows selecting the degree of coupling between the carrier fluid and the particulate phase (one-way, two-way

or four-way). In the Lagrangian formulation, the trajectories are obtained first integrating, for each particle *P* with mass m_p and velocity $\mathbf{U}_p$ (see Fig. 1), the following equation:

$$m_p \frac{d\mathbf{U}_p}{dt} = \sum \mathbf{F}_p, \tag{5}$$

where the right-hand side expresses the sum of the forces acting on the particles. This equation expresses the Lagrangian acceleration of a particle as the sum of the forces $\mathbf{F}_p$ exerted on it along its trajectory (i.e. steady state drag, virtual mass force, Basset force, pressure gradient force and body force).

Finally, once the velocities are updated, the trajectories are calculated by means of integration of the particle velocity:

$$\frac{d\mathbf{x}_p}{dt} = \mathbf{U}_p. \tag{6}$$

The two equations above describe the acceleration and the trajectories of the Lagrangian particles subject to the forces exerted by the flow. Looking in more details at the first equation, to solve it we need to define the forces acting on the particles, generally defined as functions of the carrier fluid variables at the particle's location. For this application, we neglect particle Magnus force (assuming that particle rotation is small compared to particle translation) and other forces such as added mass and Basset history terms[15], obtaining the following expression for the total force:

$$\mathbf{F}_p = \sum \mathbf{F} = drag + gravity + buoyancy. \tag{7}$$

In dilute flows, such as those of interest in this work, the dominant force acting on the particles are gravity and the drag force exerted by the fluid phase. The drag force is a function of the relative velocity of the particle, the density of the carrier phase, the particle reference area and its drag coefficient. The latter is given as a function of particle Reynolds number Re that is defined as the ratio of inertial force to friction force:

$$\mathrm{Re} = \frac{\rho_c d_p \left|\mathbf{U}_c - \mathbf{U}_p\right|}{\mu_c} = \frac{d_p \left|\mathbf{U}_c - \mathbf{U}_p\right|}{\nu_c} \tag{8}$$

where ρ_c is the density of the carrier phase, $\mathbf{U}_c$ is its velocity and μ_c and ν_c are the dynamic and kinematic viscosities, respectively, and d_p is the diameter of the particle. One common empirical correlation used for spheres is that of Schiller and Neumann (1933) and it fits well the experimental data up to a particle Reynolds number Re=1000:

$$C_D = \frac{24}{\mathrm{Re}}\left(1 + 0.15\,\mathrm{Re}^{0.687}\right). \tag{9}$$

Once the drag coefficient is known, the drag force on the particle can be evaluated as

$$\mathbf{F}_D = \frac{1}{2}\rho_c\left|\mathbf{U}_c - \mathbf{U}_p\right|\left(\mathbf{U}_c - \mathbf{U}_p\right)C_D\pi\left(\frac{d_p}{2}\right)^2. \tag{10}$$

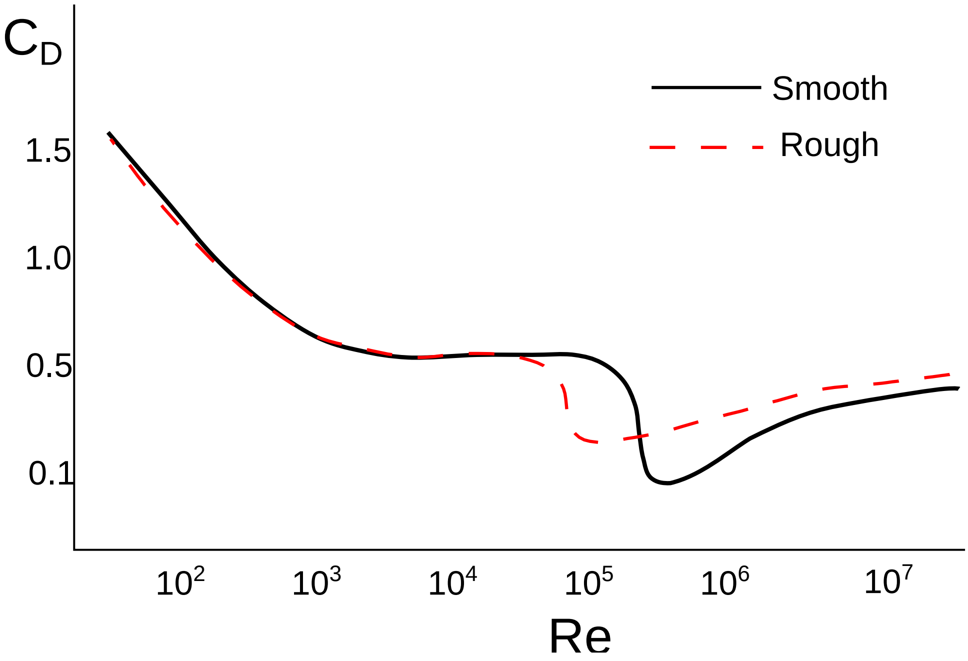

Figure 2. Drag coefficient C_D of a spherical particles as a function of Reynolds number Re, as obtained from laboratory experiments. The solid line is for a particle with smooth surface, while the dashed line is for a rough surface.

So far, we have only considered the forces of the fluid phase acting on the particles. When the effects of the presence of the particles on the fluid phase are not negligible, we have to consider and model the two-way

coupling between the phases. The force exerted by a particle P on a unit volume of fluid is proportional to the difference in particle momentum between the instant it enters (t_{in}) and leaves (t_{out}) the control volume

$$\mathbf{S}_p = -\frac{1}{V_{cell}\Delta t}\sum_P m_p\left[\left(\mathbf{U}_p\right)_{t_{out}} - \left(\mathbf{U}_p\right)_{t_{in}}\right]. \tag{11}$$

This term is then added to the momentum equation of the carrier phase. In this work, the incompressible Navier-Stokes equations are solved for a gas with volume fraction $\varepsilon = 1 - \phi_p$ where ϕ_p is the volume fraction occupied by the particles, tensor stress τ and pressure p:

$$\frac{\partial \varepsilon \mathbf{U}_c}{\partial t} + \nabla \cdot \left(\varepsilon \mathbf{U}_c \mathbf{U}_c\right) - \nabla \cdot \left(\varepsilon \tau\right) = \frac{1}{\rho_c}\left(\varepsilon \mathbf{g} - \nabla p - \mathbf{S}_p\right). \tag{12}$$

The stress tensor is given by:

$$\tau = \nu_c (\nabla \mathbf{U}_c + \left(\nabla \mathbf{U}_c\right)^T) + \frac{2}{3}\left(\nabla \cdot \mathbf{U}_c\right)\boldsymbol{\delta} \tag{13}$$

where $\boldsymbol{\delta}$ is the unit tensor and ν_c the kinematic viscosity.

Finally, the four-way coupling, considering particle-particle interactions with collisional exchanges, is modelled through a Multiphase Particle-in-Cell (MP-PIC) method[16]. In this approach, particle-particle interactions are modeled using the mean values of the particles variables interpolated on the Eulerian mesh. Collisional damping models are employed to represent the mean loss in kinetic energy, which occurs as particles collide, while collisional isotropy models are used to describe the scattering, which occurs as a result of particle collisions. The viscous stress in the forces acting on the particles is neglected. In our case, this assumption is reasonable because air viscosity is low and the main driving factors are drag and pressure gradient. The solver includes a stochastic model, which uses a time-scale to calculate the probability of a particle undergoing a collision, which randomizes its velocity.

REFERENCES

1. Ghosh, S. *et al.* How turbulence enhances coalescence of settling particles with applications to rain in clouds. *Proc. R. Soc. A Math. Phys. Eng. Sci.* **461,** 3059–3088 (2005).

2. Ishii, M. & Zuber, N. Drag coefficient and relative velocity in bubbly, droplet or particulate flows. *AIChE J.* **25,** 843–855 (1979).

3. Heymsfield, A., Giammanco, I. M. & Wright, R. Terminal velocities and kinetic energies of natural hailstones. *Geophys. Res. Lett.* **41,** 8666–8672 (2014).

4. Cheng, N.-S. Effect of Concentration on Settling Velocity of Sediment Particles. *J. Hydraul. Eng.* **123,** 728–731 (1997).

5. Folch, A. A review of tephra transport and dispersal models: Evolution, current status, and future perspectives. *J. Volcanol. Geotherm. Res.* **235–236,** 96–115 (2012).

6. Kunii, D. & Levenspiel, O. *Fluidization engineering*. (Elsevier, 2013).

7. Bonadonna, C., Ernst, G. G. J. & Sparks, R. S. J. Thickness variations and volume estimates of tephra fall deposits: the importance of particle Reynolds number. *J. Volcanol. Geotherm. Res.* **81,** 173–187 (1998).

8. Coltelli, M., Miraglia, L. & Scollo, S. Characterization of shape and terminal velocity of tephra particles erupted during the 2002 eruption of Etna volcano, Italy. *Bull. Volcanol.* **70,** 1103–1112 (2008).

9. Wilson, L. & Huang, T. C. The influence of shape on the atmospheric settling velocity of volcanic ash particles. *Earth Planet. Sci. Lett.* **44,** 311–324 (1979).

10. Dellino, P. *et al.* The analysis of the influence of pumice shape on its terminal velocity. *Geophys. Res. Lett.* **32,** 1–4 (2005).

11. Ganser, G. H. A rational approach to drag prediction of spherical and nonspherical particles. *Powder Technol.* **77,** 143–152 (1993).

12. Riley, C. M., Rose, W. I. & Bluth, G. J. S. Quantitative shape measurements of distal volcanic ash. *J. Geophys. Res.* **108,** 1–15 (2003).

13. Alfano, F., Bonadonna, C., Delmelle, P. & Costantini, L. Insights on tephra settling velocity from morphological observations. *J. Volcanol. Geotherm. Res.* **208,** 86–98 (2011).

14. Bagheri, G. H., Bonadonna, C., Manzella, I. & Vonlanthen, P. On the characterization of size and shape of irregular particles. *Powder Technol.* 141–153 (2015). doi:10.1016/j.powtec.2014.10.015

15. De'Michieli Vitturi, M., Neri, A., Esposti Ongaro, T., Lo Savio, S. & Boschi, E. Lagrangian modeling of large volcanic particles: Application to Vulcanian explosions. *J. Geophys. Res. Solid Earth* **115,** (2010).

16. O'Rourke, P. J., Zhao, P. (P) & Snider, D. A model for collisional exchange in gas/liquid/solid fluidized beds. *Chem. Eng. Sci.* **64,** 1784–1797 (2009).
